# Microbiome Depletion Increases Fentanyl Self-Administration and Alters the Striatal Proteome Through Short-Chain Fatty Acids

**DOI:** 10.1523/ENEURO.0388-23.2023

**Published:** 2024-02-09

**Authors:** Rebecca S. Hofford, Katherine R. Meckel, Elizabeth J. Wiser, Weiwei Wang, Jonathon P. Sens, Michelle Kim, Arthur Godino, TuKiet T. Lam, Drew D. Kiraly

**Affiliations:** ^1^Department of Translational Neuroscience, Wake Forest School of Medicine, Winston-Salem, NC 27101; ^2^Department of Psychiatry, Icahn School of Medicine at Mount Sinai, NewYork, NY 10029; ^3^Friedman Brain Institute, Icahn School of Medicine at Mount Sinai, NewYork, NY 10029; ^4^Nash Family Department of Neuroscience, Icahn School of Medicine at Mount Sinai, NewYork, NY 10029; ^5^Keck MS & Proteomics Resource, Yale University School of Medicine, New Haven, CT 06520; ^6^Yale/NIDA Neuroproteomics Center, Yale University School of Medicine, New Haven, CT 06520; ^7^Department of Molecular Biophysics and Biochemistry, Yale University School of Medicine, New Haven, CT 06520; ^8^Department of Psychiatry, Atrium Health Wake Forest Baptist, Winston-Salem, NC 27101

**Keywords:** fentanyl, microbiome, proteomics, self-administration

## Abstract

Opioid use disorder (OUD) is a public health crisis currently being exacerbated by increased rates of use and overdose of synthetic opioids, primarily fentanyl. Therefore, the identification of novel biomarkers and treatment strategies to reduce problematic fentanyl use and relapse to fentanyl taking is critical. In recent years, there has been a growing body of work demonstrating that the gut microbiome can serve as a potent modulator of the behavioral and transcriptional responses to both stimulants and opioids. Here, we advance this work to define how manipulations of the microbiome drive fentanyl intake and fentanyl-seeking in a translationally relevant drug self-administration model. Depletion of the microbiome of male rats with broad spectrum antibiotics leads to increased drug administration on increased fixed ratio, progressive ratio, and drug seeking after abstinence. Utilizing 16S  sequencing of microbiome contents from these animals, specific populations of bacteria from the gut microbiome correlate closely with levels of drug taking. Additionally, global proteomic analysis of the nucleus accumbens following microbiome manipulation and fentanyl administration to define how microbiome status alters the functional proteomic landscape in this key limbic substructure. These data demonstrate that an altered microbiome leads to marked changes in the synaptic proteome in response to repeated fentanyl treatment. Finally, behavioral effects of microbiome depletion are reversible by upplementation of the microbiome derived short-chain fatty acid metabolites. Taken together, these findings establish clear relevance for gut-brain signaling in models of OUD and lay foundations for further translational work in this space.

## Significance Statement

There is a growing understanding that the resident bacteria in the gut, collectively the gut microbiome, play a role in regulating brain and behavior. Previous work showed that the microbiome can mediate place preference for opioids and can alter gene expression in response to repeated drug exposure. Here, using a self-administration and drug seeking model, we see that depletion of the microbiome enhances motivation to take and seek fentanyl and shifts the dose-response curve for the drug. Additionally, we utilize quantitative global proteomics methods to define how the microbiome alters the synaptic proteome following fentanyl exposure. These studies provide important mechanistic insights to the growing literature on gut-brain signaling in substance use disorders.

## Introduction

Opioid use disorder (OUD) is a public health problem leading to tremendous morbidity and mortality. Rates of opioid overdose have been increasing rapidly (CDC, 2020) – an effect attributed at least in part to the increased presence of fentanyl in the drug supply (Zoorob, 2019). While there are currently available pharmacotherapies for opioid use disorder, they remain intolerable or ineffective for many. Understanding the neurobiological factors driving fentanyl use and relapse is a critical step in identifying novel treatments or preventative strategies to combat this epidemic.

The need for novel strategies to mitigate opioid use disorder has led to considerable research into mechanisms driving drug use outside of the central nervous system. The gut microbiome is a key regulator of health and homeostasis in diverse organ systems and disease processes. In recent years the understanding of the microbiome as a regulator of brain and behavior has increased markedly (Meckel and Kiraly, 2019; Rea et al., 2020; Lucerne et al., 2021). Recent work by our group and others has shown that alterations to the microbiome of rodents can affect cocaine (Kiraly et al., 2016; Lee et al., 2018) and opioid reward and reinforcement (Hofford et al., 2021b; Simpson et al., 2022), can influence tolerance to opioids (Kang et al., 2017; Zhang et al., 2019), and can alter cellular and molecular responses to opioids (Simpson et al., 2020; Hofford et al., 2021b). Previous work from our group measured morphine reward in mice after microbiome knockdown and found that mice with a reduced microbiome displayed reduced morphine place preference at higher doses (Hofford et al., 2021b).

There is a considerable body of work demonstrating that changes in gene expression and protein regulation in key limbic substructures is critical for the development and propagation of pathological substance use (Nestler and Lüscher, 2019). Previous work has identified that alterations in the gut microbiome can significantly alter the transcriptional landscape in the brain (Erny et al., 2015; Hoban et al., 2016, 2018; Thion et al., 2018; Chu et al., 2019). In our previous work examining the effects of the microbiome in response to morphine we observed dramatic gene expression changes in the nucleus accumbens (NAc), a brain region often implicated in the rewarding effects of drugs of abuse. Other studies examining the role of the microbiome in neuronal activation in response to opioids have also found that the microbiome changes transcriptional patterns associated with neuronal activation (Simpson et al., 2020). While this work has been critical for establishing the role of the microbiome in altering molecular responses to drugs of abuse, there have been no published studies directly assessing gut-brain effects on global protein expression changes in the brain.

This previous work from our group and others was critical in establishing a role for gut-brain signaling in models of OUD. However, the use of indirect models of opioid reward and transcriptional changes as the primary molecular readouts have left important mechanistic gaps in our understanding of this important signaling pathway. Here, we utilized a translationally relevant fentanyl self-administration model to define effects of gut microbiome depletion on fentanyl self-administration and seeking. Given the demonstrated role of the microbiome in various opioid-related behaviors, including measures of reward, it was expected that microbiome knockdown would also alter fentanyl self-administration. Crucially, we examine the effects of microbiome knockdown on several unique behavioral outputs: motivation to self-administer fentanyl under increasing fixed-ratio responding and progressive ratio, self-administration at a fixed ratio variable dose, and fentanyl-seeking after abstinence. These behavioral findings are coupled with detailed analysis of microbiome composition in relation to behavior, and quantitative proteomic analysis of the synaptic proteome of the NAc. With the inclusion of saline controls and separate groups of H_2_O and Abx rats that had equal intake or differed in their fentanyl intake, this allowed us to assess protein expression changes due to microbiome knockdown alone and due to fentanyl intake changes secondary to microbiome reduction. This work extends previous data and suggests that the gut microbiome is a mediator of fentanyl intake and seeking and identifies microbial communities, protein targets, and functional protein pathways that can be leveraged to reduce motivation to take and seek fentanyl.

## Materials and Methods

### Animals

Male Sprague-Dawley rats (Envigo) that were 11–12 weeks old were pair-housed upon arrival to the colony. Rats remained pair-housed throughout the entire experiment. All rats were kept on a 12:12 h reverse light cycle (lights on at 19:00) in a temperature and humidity-controlled vivarium. All procedures were approved by the IACUC at the Icahn School of Medicine at Mount Sinai or Wake Forest School of Medicine and all experiments conformed to the standards provided in the “Guide for the Care and Use of Laboratory Animals”. Separate groups of rats were used for Experiments 1, 2, and 3.

### Treatments

For all experiments, cages were randomly assigned to H_2_O, Abx, or Abx + SCFA so both rats in a cage received the same drink type. Drink administration started 2 weeks before the start of self-administration. Abx mixture contained 0.5 mg/ml vancomycin (Chem-Impex International #00315), 2 mg/ml neomycin (Fisher Scientific #BP266925), 0.5 mg/ml bacitracin (Research Products International #B3200025), and 1.2 µg/ml pimaricin (Infodine Chemical #7681-93-8) in H_2_O. Abx + SCFA contained the same cocktail of antibiotics plus 67.5 mM acetate, 40 mM butyrate, and 25.9 mM propionate (all SCFA from Sigma-Aldrich) dissolved in H_2_O. Control rats remained on H_2_O. Fentanyl hydrochloride was provided by the National Institute of Drug Abuse drug supply program and was dissolved in 0.9% saline.

### Fentanyl self-administration: general

Self-administration and the fentanyl-seeking test occurred in standard rat operant chambers with 2 levers, two jewel lights above each lever, a house light, and a syringe pump (Med Associates, St Albans VT). All rats were implanted with a jugular catheter under ketamine/xylazine anesthesia (100/10 mg/kg). Jugular catheters (VABR1B/22, Instech, Plymouth Meeting PA with silicone tubing, DuPont Liveo) were inserted into each rat’s jugular vein, secured to the vein with silk thread, and threaded under their skin to exit their body via a back mount. Rats were allowed to recover individually until they regained consciousness before returning to pair housing. After a one-week recovery period, rats began self-administration as described below. Sessions occurred once daily and self-administration sessions lasted 3 h during the rats’ dark phase. During self-administration, completion of the ratio requirements resulted in a 5.9 s infusion and the illumination of the light above the active lever for a 20 s time-out. Active and inactive lever placement was counterbalanced across all rats. Food was taken away from all rats 24 h before the start of acquisition and before the fentanyl-seeking test; otherwise, rats were fed 18 g standard chow/rat during active self-administration and had *ad libitum* food access before self-administration started and during abstinence.

### Fentanyl self-administration Experiment 1: increasing fixed ratio

Rats were trained to self-administer 2.5 microg/kg/infusion fentanyl or saline on a fixed-ratio 1 (FR1) schedule during acquisition which occurred once daily for 10 d. Following acquisition, fentanyl administering rats in both H_2_O and Abx groups were further divided into 2 groups- half of the rats underwent an increasing fixed ratio assessment for 6 d (referred to as H_2_O-IncFR (*n* = 6) & Abx-IncFR (*n* = 7)). This progressed to FR2 for 2 d, FR3 for 2 d, and FR5 for 2 d; the other half of the rats continued maintenance at an FR1 for 6 d (referred to as H_2_O-FR1 (*n* = 5) & Abx-FR1 (*n* = 5)). Saline-administering rats underwent increasing FR as described above (H_2_O-Sal (*n* = 7) & Abx-Sal (*n* = 7)). Immediately after the increasing FR / maintenance phase, all rats had 2 consecutive days of progressive ratio. The response requirement for each successive infusion during progressive ratio followed the formula: response ratio = [5e^(injection number×0.2)] – 5 (Richardson and Roberts, 1996). After progressive ratio, all rats underwent 2 d FR1 before 20 d of home cage abstinence. Finally, after abstinence, rats were tested on a combined context + cue fentanyl-seeking test where rats were placed in the operant boxes with both levers extended. Lever presses were recorded but had no programmed consequence for 30 min. At 30 min, the light above the active lever was illuminated for 20 s. After this, every lever press on the previously active lever resulted in illumination of the previously active lever light. Inactive lever presses continued to have no consequence. This continued for another 30 min. Twenty-four hours after their seeking test, rats were euthanized; nucleus accumbens (NAc) tissue and cecal contents were collected and flash-frozen on dry ice. Rats were dropped from the study if they had a catheter failure, or if administering fentanyl, they did not administer more than 10 infusions per day over the last 3 d of acquisition. Three H_2_O rats and two Abx rats were dropped from Experiment 1. These rats are not included in data analysis.

### Fentanyl self-administration Experiment 2: dose-response

Rats from H_2_O (*n* = 7) and Abx (*n* = 6) groups were trained to self-administer 2.5 microg/kg/infusion fentanyl on an FR1 once daily for 10 d. On the day following their last acquisition session, rats were allowed to self-administer fentanyl at the following doses: 0, 0.025, 0.25, 0.79, 7.9, and 25 microg/kg/infusion at an FR1. Rats received 2 consecutive days of every dose, but dose order across rats was randomized. Rats were dropped from the study if they had a catheter failure or did not administer more than 10 infusions per day over the last 3 d of acquisition. Two H_2_O rats and three Abx rats were dropped from Experiment 2. These rats are not included in data analysis.

### Fentanyl self-administration Experiment 3: increasing fixed ratio after short-chain fatty acid supplementation

Rats from H_2_O (*n* = 3) and Abx + SCFA (*n* = 7) groups were trained to self-administer 2.5 microgram/kg/infusion fentanyl on an FR1 once daily for 10 d. On the day following their last acquisition session, all rats underwent an increasing FR phase (2 d on FR2, 2 d on FR3, and 2 d on FR5, consecutively) followed by two, once daily sessions of progressive ratio. Rats were dropped from the study if they had a catheter failure or did not administer more than 10 infusions per day over the last 3 days of acquisition.

### 16S sequencing

Microbial DNA was isolated from the cecal contents of rats from Experiment 1 using Qiagen DNeasy PowerSoil Pro kit per kit instructions. DNA concentration was determined with a NanoDrop1000. PCR amplification was achieved using primers (341F/805R) targeting the V3 and V4 region of the 16S rRNA region of the bacterial genome and was sequenced on an Illumina NovaSeq (2 × 250 bp paired-end). Amplicons were chimera filtered, dereplicated, and paired-ends were merged using Divisive Amplicon Denoising Algorithm 2 (DADA2). These features were used to determine observed taxonomic units (OTU), defined as sequences with ≥97% similarity. OTU counts were used to determine the Shannon index of alpha diversity, the Simpson index, and principle coordinates analysis plots were generated using the Unifrac distance as an assessment of beta diversity using QIIME2 software. OTUs were identified by comparing their genetic sequences to reference bacterial genomes using SILVA (Release 132) with confidence set at 0.7.

### Sample preparation for LC–MS/MS

Tissue was collected and analyzed as described previously (Hofford et al., 2021a). Briefly, NAc tissue was lysed with a probe sonicator in solubilization buffer (RIPA buffer containing 1% proteinase and phosphatase inhibitors). Lysate was centrifuged at 14K rpm for 10 min at 4°C in a tabletop centrifuge to pellet cellular debris, and supernatant was protein precipitated using a methanol chloroform method. Proteins pellet (50 µg) was resuspended in 50uL solubilization buffer (8M urea in 0.4M ammonium bicarbonate), reduced with 5 µl of 45 mM dithiothreitol (DTT) and incubated at 37°C for 30 min. They were then alkylated with 5 µl of 100 mM iodoacetamide (IAN) and incubated in the dark at room temperature for 30 min. After diluting with water to bring urea concentration to 2 M, sequencing-grade Lys-C (New England Biolabs, Ipswich, MA, USA) was added at a weight ratio of 1:50 (Lys-C/protein) and incubated at 37°C overnight. Trypsin (Promega, Madison, WI, USA) was then added at similar weight ratio and incubated for 4 h at 37°C. The digested samples were then acidified with 0.1% formic acid, desalted using C18 spin columns (The Nest Group, Inc., Southborough, MA, USA), and dried in a rotary evaporator. The samples were resuspended in 0.2% trifluoroacetic acid (TFA) and 2% acetonitrile (ACN) in water prior to LC–MS/MS analysis.

### Data-independent acquisition (DIA)

DIA LC–MS/MS was performed using a nanoACQUITY UPLC system (Waters Corporation, Milford, MA, USA) connected to a Q-Exactive HFX (ThermoFisher Scientific, San Jose, CA, USA) mass spectrometer. After injection, the samples were loaded into a trapping column (nanoEase M/Z Symmetry C18 Trap column, 180 µm × 20 mm) for 3 min at a flow rate of 10 µl/min and separated with a C18 column (nanoEase M/Z column Peptide BEH C18, 75 µm × 250 mm). The compositions of mobile phases A and B were 0.1% formic acid in water and 0.1% formic acid in ACN, respectively. The peptides were separated and eluted with a gradient extending from 6 to 25% mobile phase B in 98 min and then to 85% mobile phase B in additional 5 min at a flow rate of 300 nl/min and a column temperature of 37°C. Column regeneration and up to three blank injections were carried out in between all sample injections. The data were acquired with the Q-Exactive HFX mass spectrometer operating in a data-independent acquisition mode with an isolation window width of 10 m/z. The full scan was performed in the range of 400–1,000 m/z with “Use Quadrupole Isolation” enabled at an Orbitrap resolution of 120,000 at 200 m/z and automatic gain control (AGC) target value of 3 × 10^6^. Fragment ions from each peptide MS^2^ were generated in the C-trap with higher-energy collision dissociation (HCD) at a normalized collision energy of 28% and detected in the Orbitrap at a resolution of 30,000.

DIA spectra were searched against a *Rattus norvegicus* brain proteome fractionated spectral library generated from DDA LC MS/MS spectra (collected from the same Q-Exactive HFX mass spectrometer) using Scaffold DIA software v. 2.2.0 (Proteome Software, Portland, OR, USA). Within Scaffold DIA, raw files were first converted to the mzML format using ProteoWizard v. 3.0.11748. The samples were then aligned by retention time and individually searched with a mass tolerance of 10 ppm and a fragment mass tolerance of 10 ppm. The data acquisition type was set to “Non-Overlapping DIA”, and the maximum missed cleavages was set to 2. Fixed modifications included carbamidomethylation of cysteine residues (+57.02). Dynamic modifications included phosphorylation of serine, threonine, and tyrosine (+79.96), deamination of asparagine and glutamine (+0.98), oxidation of methionine and proline (+15.99), and acetylation of lysine (+42.01). Peptides with charge states between 2 and 4 and 6–30 amino acids in length were considered for quantitation, and the resulting peptides were filtered by Percolator v. 3.01 at a threshold FDR of 0.01. Peptide quantification was performed by EncyclopeDIA v. 0.6.12 and six of the highest quality fragment ions were selected for quantitation. Proteins containing redundant peptides were grouped to satisfy the principles of parsimony, and proteins were filtered at a threshold of two peptides per protein and an FDR of 1%. The mass spectrometry proteomics data have been deposited to the ProteomeXchange Consortium via the PRIDE (Perez-Riverol et al., 2022) partner repository with the dataset identifier PXD035810.

### Pathway analysis and visualization of protein networks

**Figure 1.**
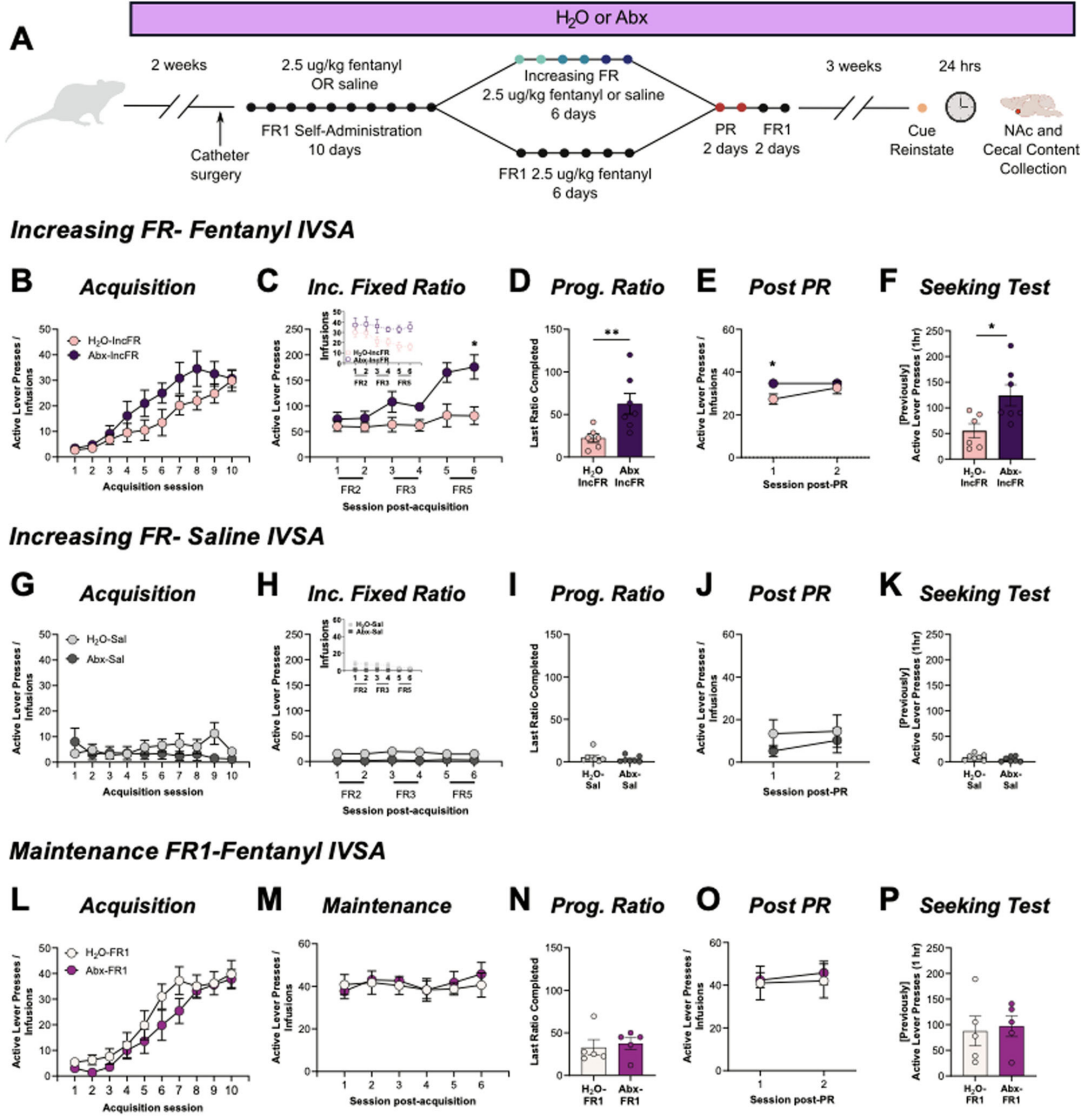
Abx increases motivation to self-administer fentanyl. ***A***, Experimental timeline for Experiment 1. ***B***, H_2_O-IncFR and Abx-IncFR rats acquired FR1 fentanyl administration at equal rates. ***C***, At increasing FR requirements Abx caused enhanced responding. Inset shows number of infusions earned. ***D***, Abx-IncFR rats had higher breakpoints on a progressive ratio task. ***E***, Return to FR1 normalized responding between groups over two sessions. ***F***, Abx treatment increased fentanyl-seeking after withdrawal in Abx-IncFR rats. ***G–K***, Abx did not affect self-administration of saline. ***L–P***, As a control, another group of rats was maintained on FR1 responding throughout (H_2_O-FR1, Abx-FR1), and there were no differences in acquisition of self-administration, maintenance of self-administration, progressive ratio, post-PR FR1 responding, or fentanyl-seeking. Data presented as means ± SEM. * *p* < 0.05, ** *p* < 0.01. Full statistical results for active and inactive lever presses are included in Tables 1 and 2.

**Figure 2.**
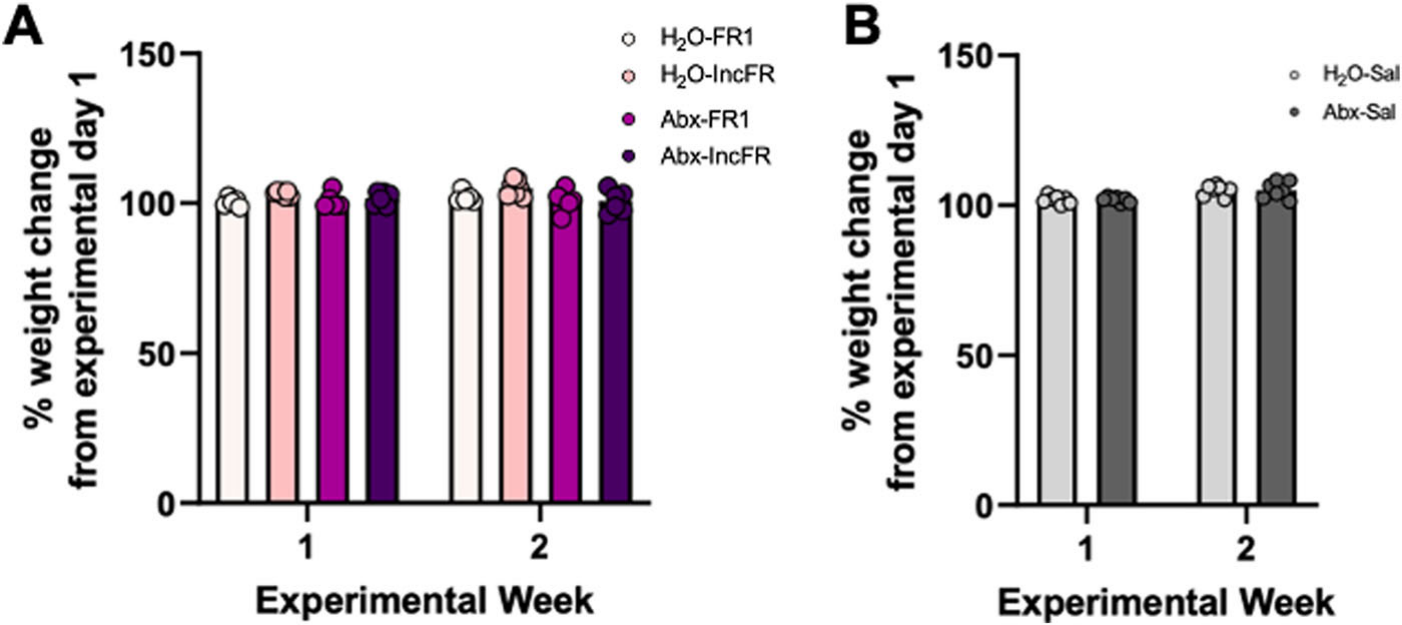
Abx treatment does not have effects on body weight. Changes in bodyweight for all groups for the first two weeks of all groups was measured in fentanyl (***A***) and saline (***B***) administering rats. There were no main effects or interactions seen due to Abx treatment.

**Figure 3.**
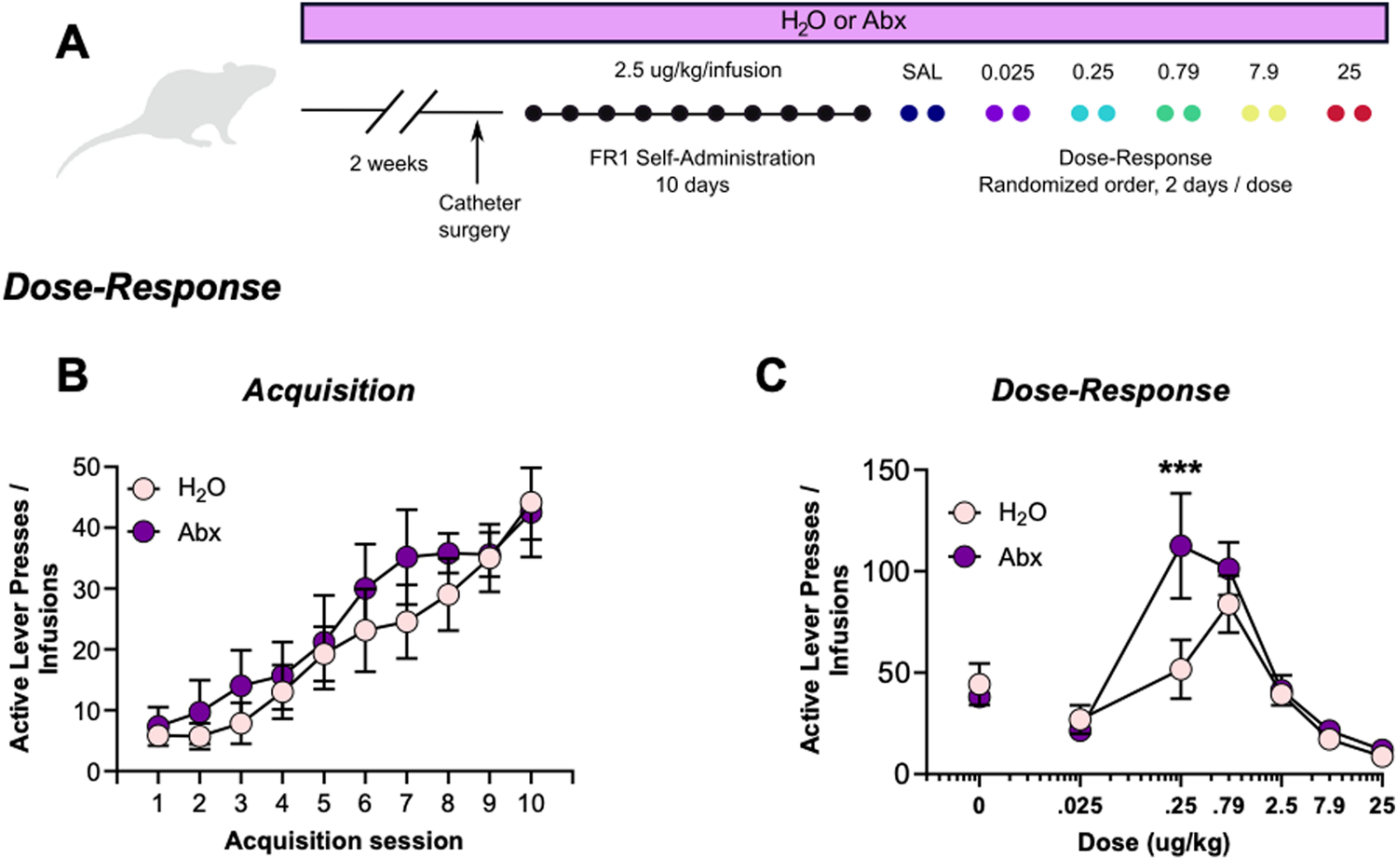
Abx shifts the dose-response curve. ***A***, Experimental timeline for Experiment 2. ***B***, Abx did not affect acquisition of self-administration. ***C***, However, there was a significant interaction between dose and Abx treatment; post hoc test indicated a significant difference between groups at the 0.25 microg/kg/infusion dose. Data presented as means ± SEM. ***p < 0.001. Full statistical results for active and inactive lever presses are included in Tables 1 and 2.

Proteins were excluded from analysis if they were not detected in >50% of all samples irrespective of treatment. Pairwise comparisons of the Log_10_ median intensity of every remaining protein and protein group were made using Scaffold DIA proteomics analysis software (http://www.proteomesoftware.com/products/dia/). Technical replicates were treated as independent samples and proteins were considered significantly differentially regulated when FDR corrected *p* < 0.1. All groups were compared to H_2_O-Sal to allow for inferences across comparisons. Significantly upregulated and downregulated proteins were separately uploaded into the open source pathway analysis software package G:Profiler (Raudvere et al., 2019) (https://biit.cs.ut.ee/gprofiler/gost) to identify significantly enriched Gene Ontologies (GO) and Kyoto Encyclopedia of Genes and Genomes (KEGG) pathways. Enrichr (Chen et al., 2013; Kuleshov et al., 2016) (https://maayanlab.cloud/Enrichr/) was used to identify upstream predicted transcription factors using the “ENCODE and CheA consensus transcription factors from Chip-X” list using an FDR corrected *p* < 0.05. Additionally, all significantly regulated proteins were uploaded to Ingenuity Pathway Analysis (IPA) for analysis of pathway directionality between comparisons using an FDR corrected *p* < 0.05. A list of shared upregulated proteins between fentanyl self-administration groups that excluded proteins upregulated in Abx Sal was generated using DeepVenn (Hulsen et al., 2008) (http://www.deepvenn.com/); these proteins were separately uploaded into the STRING database (Szklarczyk et al., 2019) (https://string-db.org/). Cytoscape with STRING add-in was used for visualization of protein-protein interaction within the IPA pathway “synaptogenesis signaling pathway”. Given that G:Profiler, Enrichr, STRING, and IPA use gene names for identifying pathways, all protein names were first converted to gene names prior to analysis using the Uniprot database (https://www.uniprot.org/id-mapping). Portions of Figures 1, 2, 3, and 5 were made with Biorender.com with full permission to publish.

### Experimental design and statistical analyses

Since the primary goal of this experiment was to compare the effects of microbiome knockdown on fentanyl self-administration under different behavioral conditions, *a priori* comparisons were used to compare H_2_O and Abx groups directly to each other if they were administering the same drug (fentanyl or saline) and they underwent the same cycle of self-administration phases (e.g., Experiment 1 increasing FR or Experiment 1 maintenance). Only active and inactive lever presses during the non-time out period were graphed and used in data analyses. Active and inactive lever presses during acquisition, increasing fixed ratio, maintenance, and FR1 after progressive ratio were analyzed separately using linear mixed effects with drink type as a fixed between-subjects factor and session or dose as a fixed within-subjects factor. Active and inactive lever presses during the dose-response were analyzed with a two-way ANOVA with drink type as a fixed between-subjects factor and dose as a fixed within-subjects factor. Progressive ratio breakpoints were averaged over two sessions; for experiment 1 average breakpoint and lever presses during the fentanyl-seeking test were analyzed using Mann-Whitney tests between H_2_O and Abx for each set of rats administering the same drug and undergoing the same cycle of self-administration phases. When collapsing data between experiment 1 and 3, Kruskal-Wallis test was used to determine differences in breakpoints and inactive lever presses during progressive ratio. In the presence of significant interactions, differences between H_2_O and Abx at all sessions or doses were analyzed using Holm-Sidak correction.

The diversity measures of the microbiome (number of OTUs and the Simpson index) were analyzed using two-way ANOVA with drink type and self-administration paradigm as fixed between-subjects factors. Fold change of phylum abundance was analyzed using multiple *t*-tests comparing H_2_O-Sal to each fentanyl administering group and FDR correction was applied to control for multiple comparisons. For correlations: genera were chosen if *R*^2^ > 0.5 and *p* < 0.05; *t*-tests were run on relative abundance of these genera between fentanyl administering H_2_O and Abx rats from Experiment 1 collapsed across self-administration paradigm (IncFR and FR1). For all analyses: Greenhouse-Geiser corrections were applied when appropriate and statistical outliers were removed when significantly more than two standard deviations from the rest of the data points in that group.

## Results

### Microbiome knockdown via Abx enhances motivation for fentanyl and shifts the peak of the dose-response curve

To investigate the consequence of microbiome knockdown on fentanyl intake and motivation, rats in Experiment 1 were treated with antibiotics (Abx) to reduce the microbiome or maintained on control water (Fig. 1*A*). As expected, microbiome knockdown did not alter saline intake (session: *F*_(1.83, 22.01)_ = 1.01, *p* = 0.38; drink: *F*_(1,12)_ = 0.57, *p* = 0.47, interaction: *F*_(9,108)_ = 2.73, *p* = 0.007, but no significant pairwise comparison were identified) and did not affect acquisition of fentanyl self-administration when trained at an FR1 (main effect of session only *F*_(1.62,17.66)_ = 29.9, *p* < 0.0001; drink: *F*_(1,11)_ = 2.07, *p* = 0.18; interaction: *F*_(9, 98)_ = 1.361, *p* = 0.22). However, when rats self-administered at higher fixed ratios, Abx-IncFR rats increased effort to obtain fentanyl as it became harder to earn (Fig. 1*C*, session: *F*_(2.06,22.19)_ = 25.51, *p* < 0.0001; drink: *F*_(1,11)_ = 5.39, *p* = 0.04; and interaction: *F*_(5,54)_ = 9.94, *p* < 0.0001), and had higher breakpoints on a progressive ratio (PR) task (Fig. 1*D*, *U* = 2.5, *p* = 0.0058).

**Table 2.** Statistical analysis of inactive lever pressing

Group	Phase	Level	Statistic	*p* value	Post-hoc
Experiment 1: IncFR (H_2_O, Abx)	Acquisition	Session	*F*_(2.772, 30.49)_ = 0.8443	0.4725	
		Drink	*F*_(1, 11)_ = 0.695	0.422	
		Interaction	*F*_(9, 99)_ = 0.8152	0.6034	
Experiment 1: Sal (H_2_O, Abx)	Acquisition	Session	*F*_(2.595, 31.14)_ = 1.039	0.381	
		Drink	*F*_(1, 12)_ = 0.657	0.4334	
		Interaction	*F*_(9, 108)_ = 0.3048	0.9718	
Experiment 1: FR1 (H_2_O, Abx)	Acquisition	Session	*F*_(1.88, 14.19)_ = 1.23	0.3193	
		Drink	*F*_(1, 8)_ = 2.36	0.1632	
		Interaction	*F*_(9, 68)_ = 0.996	0.4525	
Experiment 1: IncFR (H_2_O, Abx)	Increasing FR	Session	*F*_(1.663, 17.96)_ = 1.072	0.3515	
		Drink	*F*_(1, 11)_ = 1.934	0.1918	
		Interaction	*F*_(5, 54)_ = 0.4437	0.816	
Experiment 1: Sal (H_2_O, Abx)	Increasing FR	Session	*F*_(2.457, 29.48)_ = 0.7523	0.5055	
		Drink	*F*_(1, 12)_ = 1.746	0.2111	
		Interaction	*F*_(5, 60)_ = 0.419	0.8337	
Experiment 1: FR1 (H_2_O, Abx)	Maintenance	Session	*F*_(2.12, 14.45)_ = 1.07	0.3724	
		Drink	*F*_(1, 8)_ = 1.500	0.2554	
		Interaction	*F*_(5, 34)_ = 2.043	0.0973	
Experiment 1: IncFR (H_2_O, Abx)	Progressive Ratio		*U* = 9.0	0.0904	
Experiment 1: Sal (H_2_O, Abx)	Progressive Ratio		*U* = 16.0	0.2966	
Experiment 1: FR1 (H_2_O, Abx)	Progressive Ratio		*U* = 10.0	0.6349	
Experiment 1: IncFR (H_2_O, Abx)	Post-PR	Session	*F*_(1, 10)_ = 1.868	0.2016	
		Drink	*F*_(1, 10)_ = 2.543	0.1418	
		Interaction	*F*_(1, 10)_ = 2.229	0.1663	
Experiment 1: Sal (H_2_O, Abx)	Post-PR	Session	*F*_(1, 12)_ = 0.3650	0.557	
		Drink	*F*_(1, 12)_ = 0.1647	0.692	
		Interaction	*F*_(1, 12)_ = 1.848	0.119	
Experiment 1: FR1 (H_2_O, Abx)	Post-PR	Session	*F*_(1, 7)_ = 0.4227	0.5363	
		Drink	*F*_(1, 7)_ = 1.238	0.3026	
		Interaction	*F*_(1, 7)_ = 2.671	0.1462	
Experiment 1: IncFR (H_2_O, Abx)	Fentanyl-Seeking		*t*_(11)_ = 0.2238	0.827	
Experiment 1: Sal (H_2_O, Abx)	Fentanyl-Seeking		*t*_(12)_ = 1.812	0.095	
Experiment 1: FR1 (H_2_O, Abx)	Fentanyl-Seeking		*t*_(7)_ = 1.098	0.3085	
Experiment 2 (H_2_O, Abx)	Acquisition	Session	*F*_(2.453, 24.80)_ = 4.071	0.0231*	No significant pairwise comparisons
		Drink	*F*_(1, 11)_ = 0.7137	0.4162	
		Interaction	*F*_(9, 91)_ = 2.521	0.0126*	
Experiment 2 (H_2_O, Abx)		Dose	*F*_(2.591, 27.20)_ = 1.017	0.3918	
	Dose-Response	Drink	*F*_(1, 11)_ = 0.1931	0.6689	
	Lever Press	Interaction	*F*_(6, 63)_ = 0.9105	0.4933	
Experiment 3 (H_2_O, Abx + SCFA)	Acquisition	Session	*F*_(3.062, 12.82)_ = 1.364	0.2777	
		Drink	*F*_(1, 8)_ = 23.08	0.0013**	
		Interaction	*F*_(9, 70)_ = 1.488	0.1696	
Experiment 3 (H_2_O, Abx + SCFA)	Increasing FR	Session	*F*_(5.24, 20.32)_ = 3.492	0.2777	
		Drink	*F*_(1, 8)_ = 0.0028	0.959	
		Interaction	*F*_(5, 40)_ = 1.75	0.1454	
Experiment 3 (H_2_O, Abx + SCFA)	Progressive Ratio		*U* = 7.0	0.5167	
Comparing Exp 1 and Exp 3 : H_2_O only (Exp 1 H_2_O, Exp 3 H_2_O)	Increasing FR	Session	*F*_(2.552,17.87)_ = 4.994	0.0137*	*p* < 0.05 Day 6
		Experiment	*F*_(1,7)_ = 4.386	0.0745	
		Interaction	*F*_(5,35)_ = 5.025	0.0014**	
Comparing Exp 1 and Exp 3 : H_2_O only (Exp 1 H_2_O, Exp 3 H_2_O)	Progressive Ratio		*U* = 3.0	0.1667	
Exp 1 and Exp 3 (H_2_O, Abx, Abx + SCFA)	Increasing FR	Session	*F*_(2.298,45.50)_ = 2.372	0.0975	
		Drink	*F*_(2,20)_ = 0.7468	0.4867	
		Interaction	*F*_(10,99)_ = 0.945	0.4961	
Exp 1 and Exp 3 (H_2_O, Abx, Abx + SCFA)	Progressive Ratio		H = 1.075	0.5842	

Detailed statistical analysis for inactive lever pressing in Experiments 1, 2 and 3.

*p ≤ 0.05; **p ≤ 0.01.

Rats self-administering saline had no difference in active lever presses during acquisition, increased fixed ratio, progressive ratio, post-progressive ratio, or drug seeking tests (Fig. 1*G–K*).

Since the fixed ratio requirements increased over time (FR2/FR3/FR5) and opioid tolerance can change over time in a microbiome-dependent manner (Kang et al., 2017; Zhang et al., 2019), a separate group of fentanyl-administering rats were kept at FR1 (referred to as H_2_O-FR1 and Abx-FR1) and were run concurrently (Fig. 1*A*, bottom). When maintained at FR1, microbiome knockdown had no effect on acquisition of fentanyl self-administration (Fig. 1*L*, session: *F*_(2.02,15.71)_ = 47.33, *p* < 0.0001; drink: *F*_(1,8)_ = 1.18, *p* = 0.31; interaction: *F*_(9,70)_ = 0.98, *p* = 0.47), maintenance of FR1 responding over time (Fig. 1*M*, session: *F*_(1.98,15.42)_ = 0.57, *p* = 0.57; drink: *F*_(1,8)_ = 0.11, *p* = 0.74; and interaction: *F*_(5,39)_ = 0.36, *p* = 0.87), or with PR testing (Fig. 1*N*, *U* = 9.0, *p* = 0.516). Thus, Abx-induced enhancement in responding for fentanyl during the increasing FR phase represents an increase in motivation for fentanyl instead of altered tolerance or a shifting microbiome.

Following PR testing, all rats were re-stabilized on FR1 (Fig. 1*E*,*J*,*O*) before undergoing 20 days of abstinence. After abstinence, Abx-IncFR rats showed markedly higher levels of fentanyl-seeking during a context and cue fentanyl-seeking task (Fig. 1*F*, *U* = 6.0, *p* = 0.035), but this was not seen with Abx-FR1 rats (Fig. 1*P*, *U* = 10.5, *p* = 0.7302).

To accurately assess behavioral changes in these assays, it is critical to ensure that our intervention of depleting the microbiome with antibiotics is not affecting the health and development of the rats. To check this we assessed changes in bodyweight for the first two weeks in all animals in Experiment 1. There were no significant effects on body weight changes between any fentanyl groups as shown in Figure 2*A* - H_2_O-FR1, H_2_O-IncFR, Abx-FR1, and Abx-IncFR (mixed effects, group: *F*_(3,19)_ = 3.04, *p* = 0.054; experimental week: *F*_(1,19)_ = 1.36, *p* = 0.26; interaction: *F*_(3,19)_ = 2.48, *p* = 0.09). Similarly, no differences were found with Abx treatment in saline administering rats as in Figure 2*B* though a very modest effect of experimental week was noted (mixed effects, group: *F*_(1,12)_ = 0.005, *p* = 0.95; experimental week: *F*_(1,12)_ *=* 21.33, ***p = 0.0006; interaction: *F*_(1,12)_ = 0.002, *p* = 0.96).

Since Abx produced no changes in acquisition at 2.5 microg/kg/infusion but led to differences in motivation to administer at higher FRs, we considered the possibility that microbiome disruption causes a shift in the dose-response curve. To test this, Experiment 2 utilized a separate group of rats that were treated and trained to administer fentanyl as described above (Fig. 3*A*). Like previously observed, Abx did not alter the rate of acquisition (Fig. 3*B*, session: *F*_(1.59,16.93)_ = 27.23, *p* < 0.0001; drink: *F*_(1,11)_ = 0.56, *p* = 0.47; interaction: *F*_(9,96)_ = 0.55, *p* = 0.83). However, there was a significant interaction between treatment and dose during assessment of their dose-response curves (Fig. 3*C*, interaction: *F*_(5,66)_ = 2.61, *p* = 0.03; dose: *F*_(5,66)_ = 21.09, *p* < 0.0001; drink: *F*_(1,66)_ = 4.99, *p* = 0.03), demonstrating a leftward and upward shift of the peak of the dose-response curve after Abx. Additionally, a significant post-hoc difference between groups at the 0.25 microg/kg dose (*p* = 0.0008). Full active lever and inactive lever statistics included in Tables 1 and 2.

**Table 1.** Statistical analysis of active lever pressing

Group	Phase	Level	Statistic	*p* value	Post-hoc
Experiment 1: IncFR (H_2_O, Abx)	Acquisition	Session	*F*_(1.622, 17.66)_ = 29.9	<0.0001****	
		Drink	*F*_(1, 11)_ _=_ 2.07	0.179	
		Interaction	*F*_(9, 98)_ = 1.361	0.2162	
Experiment 1: Sal (H_2_O, Abx)	Acquisition	Session	*F*_(1.83, 22.01)_ = 1.005	0.3759	No significant pairwise comparisons
		Drink	*F*_(1, 12)_ = 0.565	0.47	
		Interaction	*F*_(9, 108)_ = 2.73	0.0066**	
Experiment 1: FR1 (H_2_O, Abx)	Acquisition	Session	*F*_(2.020, 15.71)_ = 47.33	<0.0001****	
		Drink	*F*_(1, 8)_ = 1.183	0.3085	
		Interaction	*F*_(9, 70)_ = 0.9778	0.4659	
Experiment 1: IncFR (H_2_O, Abx)	Increasing FR	Session	*F*_(2.055, 22.19)_ = 25.51	<0.0001****	*p* < 0.05: day 6
		Drink	*F*_(1, 11)_ = 5.391	0.0404*	
		Interaction	*F*_(5, 54)_ = 9.942	<0.0001****	
Experiment 1: Sal (H_2_O, Abx)	Increasing FR	Session	*F*_(2.92, 35.04)_ = 0.691	0.56	
		Drink	*F*_(1, 12)_ = 4.87	0.0475*	
		Interaction	*F*_(5, 60)_ = 0.6642	0.652	
Experiment 1: FR1 (H_2_O, Abx)	Maintenance	Session	*F*_(1.977, 15.42)_ = 0.5725	0.5738	
		Drink	*F*_(1, 8)_ = 0.1149	0.7433	
		Interaction	*F*_(5, 39)_ = 0.3583	0.8737	
Experiment 1: IncFR (H_2_O, Abx)	Progressive Ratio		*U* = 2.5	0.0058**	
Experiment 1: Sal (H_2_O, Abx)	Progressive Ratio		*U* = 15.5	0.277	
Experiment 1: FR1 (H_2_O, Abx)	Progressive Ratio		*U* = 9.0	0.516	
Experiment 1: IncFR (H_2_O, Abx)	Post-PR	Session	*F*_(1, 10)_ = 10.24	0.0095**	*p* < 0.05: day 1
		Drink	*F*_(1, 10)_ = 2.647	0.1348	
		Interaction	*F*_(1, 10)_ = 10.24	0.0095**	
Experiment 1: Sal (H_2_O, Abx)	Post-PR	Session	*F*_(1, 12)_ = 2.682	0.1274	
		Drink	*F*_(1, 12)_ = 0.5698	0.4649	
		Interaction	*F*_(1, 12)_ = 1.058	0.3241	
Experiment 1: FR1 (H_2_O, Abx)	Post-PR	Session	*F*_(1, 8)_ = 1.646	0.2354	
		Drink	*F*_(1, 8)_ = 0.08304	0.7805	
		Interaction	*F*_(1, 8)_ = 0.4898	0.5039	
Experiment 1: IncFR (H_2_O, Abx)	Fentanyl-Seeking		*U* = 6.0	0.035*	
Experiment 1: Sal (H_2_O, Abx)	Fentanyl-Seeking		*U* = 12.5	0.1434	
Experiment 1: FR1 (H_2_O, Abx)	Fentanyl-Seeking		*U* = 10.5	0.0903	
Experiment 2 (H_2_O, Abx)	Acquisition	Session	*F*_(1.587, 16.93)_ = 27.23	<0.0001****	
		Drink	*F*_(1, 11)_ = 0.5607	0.4697	
		Interaction	*F*_(9, 96)_ = 0.5534	0.8317	
Experiment 2 (H_2_O, Abx)	Dose-Response Lever Press	Dose	*F*_(5, 66)_ = 21.09	<0.0001****	*p* < 0.001: 0.25 microg/kg
		Drink	*F*_(1, 66)_ = 4.999	0.0328*	
		Interaction	*F*_(5, 66)_ = 2.605	0.0301*	
Experiment 3 (H_2_O, Abx + SCFA)	Acquisition	Session	*F*_(2.295,17.85)_ = 7.96	0.0025**	
		Drink	*F*_(1, 8)_ = 0.0099	0.923	
		Interaction	*F*_(9, 70)_ = 0.1723	0.9962	
Experiment 3 (H_2_O, Abx + SCFA)	Increasing FR	Session	*F*_(1.59,12.72)_ = 5.29	0.0267*	
		Drink	*F*_(1, 8)_ = 0.4836	0.507	
		Interaction	*F*_(5, 40)_ = 2.21	0.0703	
Experiment 3 (H_2_O, Abx + SCFA)	Progressive Ratio		*U* = 8.5	0.7167	
Comparing Exp 1 and Exp 3 : H_2_O only (Exp 1 H_2_O, Exp 3 H_2_O)	Increasing FR	Session	*F*_(1.696,11.87)_ = 5.286	0.0266*	
		Experiment	*F*_(1,7)_ = 0.264	0.6232	
		Interaction	*F*_(5,35)_ = 1.780	0.1426	
Comparing Exp 1 and Exp 3 : H_2_O only (Exp 1 H_2_O, Exp 3 H_2_O)	Progressive Ratio		*U* = 8.0	0.8452	
Exp 1 and Exp 3 (H_2_O, Abx, Abx + SCFA)	Increasing FR	Session	*F*_(1.791,35.47)_ = 21.07	<0.0001****	*p* < 0.05: Day 4 H_2_O vs Abx and Abx vs Abx + SCFA
		Experiment	*F*_(2,20)_ = 3.644	0.0447	*p* < 0.05: Day 5 H_2_O vs Abx and Abx vs Abx + SCFA
		Interaction	*F*_(10,99)_ = 3.826	0.0002***	*p* < 0.05: Day 6 H_2_O vs Abx and *p* < 0.01 Abx vs Abx + SCFA
Exp 1 and Exp 3 (H_2_O, Abx, Abx + SCFA)	Progressive Ratio		H = 10.38	0.0056**	*p* *<* 0.05: H_2_O vs Abx, *p* *<* 0.01: Abx vs Abx + SCFA

Detailed statistical analysis for active lever pressing in Experiments 1, 2 and 3.

*p ≤ 0.05; **p ≤ 0.01; ***p ≤ 0.001; ****p ≤ 0.0001.

### Fentanyl intake during increasing FR and maintenance is negatively correlated with several bacterial genera

**Figure 4.**
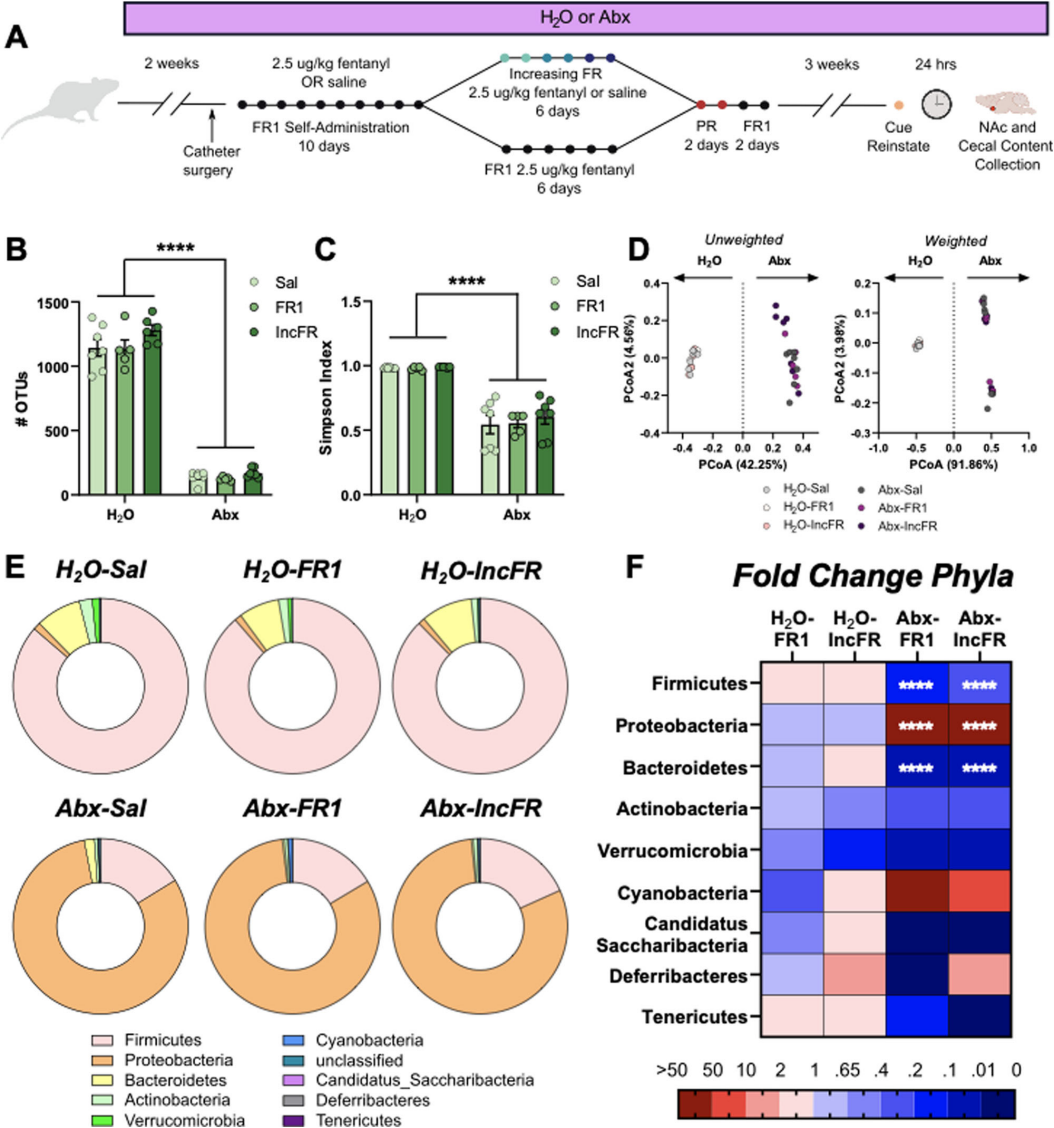
Abx alters the microbiome. ***A***, Experimental timeline for Experiment 1 and microbiome collection. Abx reduced microbiome diversity as measured by the number of OTUs (***B***) and Simpson (***C***) indices. (***D***) The microbiomes of H_2_O and Abx rats differed markedly when assessed with either an unweighted (left) or weighted (right) Unifrac dissimilarity matrix. Microbial composition was driven primarily by Abx treatment. ***E***, Donut plots of bacterial phyla abundance in all six groups of rats. ***F***, Heatmap of phyla abundance as fold change from H2O Sal in fentanyl-administering groups of rats. ****p < 0.0001. Data presented as means ± SEM. Full statistical results for diversity indices and phylum abundance are included in Extended Data Tables 4-1 and 4-2.

We next performed detailed analysis of changes to the microbiome caused by Abx and fentanyl self-administration using 16S sequencing of cecal contents from rats in Experiment 1 (Fig. 4*A*). As expected, Abx drastically reduced indices of alpha diversity (Fig. 4*B,C*, Number of OTUs: drink: *F*_(1, 31)_ = 865.0, *p* < 0.0001; self-administration paradigm: *F*_(2, 31)_ = 2.93, *p* = 0.0683; interaction: *F*_(2, 31)_ = 0.9925, *p* = 0.3821; Simpson index drink: *F*_(1, 31)_ = 129.3, *p* < 0.0001; self-administration paradigm: *F*_(2, 31)_ = 0.3608, *p* = 0.7; interaction: *F*_(2, 31)_ = 0.2261, *p* = 0.80). Additionally, as observed in our prior study utilizing morphine (Hofford et al., 2021b), we found that Abx treatment drove the largest separation of samples using either an unweighted or weighted Unifrac dissimilarity matrix. Additionally, opioid history did not significantly alter diversity metrics of the microbiome - there was no visible separation of fentanyl and saline samples (Fig. 4*D*). Visualization of phyla abundance across groups indicated that relative abundance of Firmicutes and Bacteroidetes were reduced and levels of Proteobacteria were expanded after Abx (Fig. 4*E,F*, full statistics on phyla and genera abundance included in Extended Data Tables 4-1 and 4-2).

10.1523/ENEURO.0388-23.2023.t4-1 Table 4-1 Number of OTUs, Simpson index, and genus abundance statistics for 16s sequencing analyses. Download Table 4-1, XLS file.

10.1523/ENEURO.0388-23.2023.t4-2 Table 4-2 Full phylum abundance list and statistics for 16s sequencing analyses. Download Table 4-2, XLS file.

**Figure 5.**
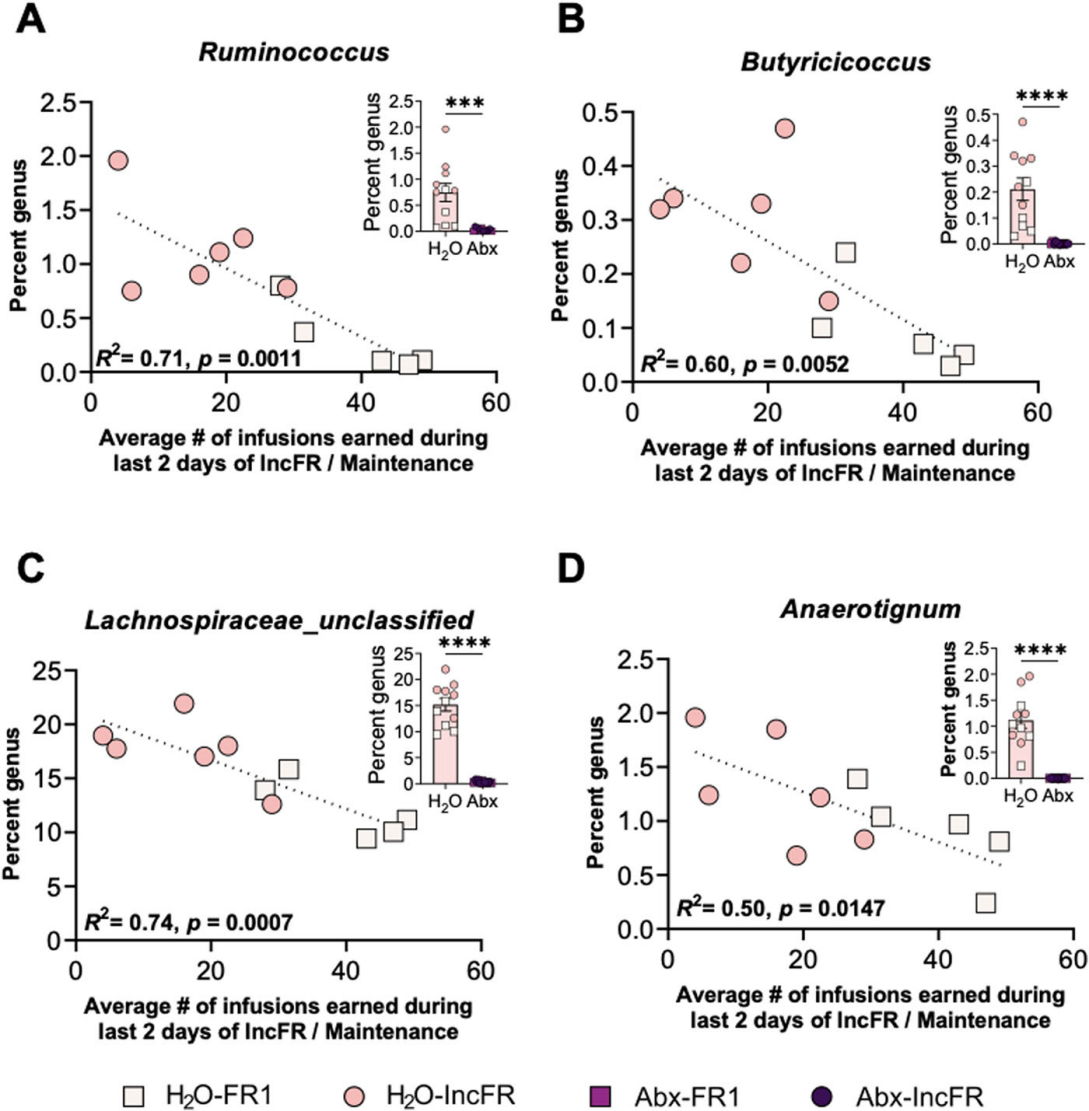
Abundance of several genera correlate with fentanyl intake in rats with an intact microbiome. Correlation plots of H_2_O fentanyl rats’ fentanyl intake with relative abundance of (***A***) *Ruminococcus*, (***B***) *Butyricicoccus*, (***C***) *Lachnospiraceae_unclassified,* and (***D***) *Anaerotignum*. Insets: percent abundance of each genus in H_2_O and Abx groups, collapsed across administration paradigm (FR1 and IncFR combined). ***p < 0.001, ***p < 0.0001. Data presented as means ± SEM. Full list of correlations between fentanyl intake and genus abundance are in Extended Data Table 5-1.

Next, we examined the relationship between fentanyl intake and genus abundance within our H_2_O groups (H_2_O-FR1 and H_2_O-IncFR). Abundance of *Ruminococcus, Butyricicoccus, Lachnospiracae_unclassified,* and *Anaerotignum* negatively correlated with fentanyl intake during the last 2 d of fentanyl increasing FR or maintenance (Fig. 5*A–D*). Interestingly, the abundance of these four genera was markedly diminished by Abx treatment (Fig. 5*A–D*, insets), suggesting that low levels of these bacteria could enhance motivation for and sensitivity to the reinforcing properties of fentanyl observed here after microbiome knockdown. Correlation statistics included in Extended Data Table 5-1.

10.1523/ENEURO.0388-23.2023.t5-1 Table 5-1 Full correlation matrix between fentanyl intake and genus abundance. Download Table 5-1, XLS file.

### Microbiome knockdown alters global protein expression after a fentanyl-seeking task

**Figure 6.**
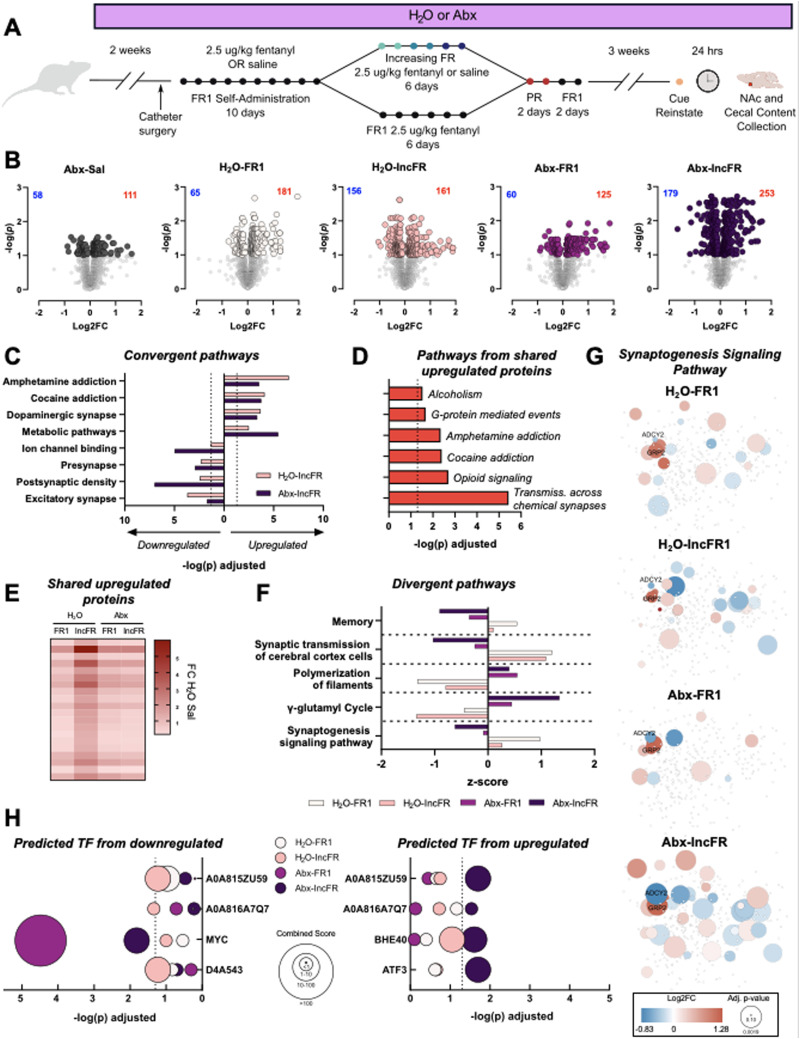
Microbiome knockdown alters the nucleus accumbens proteome. ***A***, Experimental timeline for Experiment 1 and NAc collection. ***B***, Volcano plots depicting protein expression changes in rats compared to H_2_O Sal. Colored points are significantly regulated proteins (FDR-corrected *p* < 0.1). ***C***, Select pathways that are predicted to be regulated in the same direction in H_2_O-IncFR and Abx-IncFR groups. Dotted line at 1.3 indicates significance (FDR-corrected *p* < 0.05). ***D***, Predicted pathways from proteins upregulated in all fentanyl-administering groups but not present in Abx-Sal. ***E***, Heatmap of fold change protein expression of overlapping upregulated proteins in fentanyl-administering rats. ***F***, Select pathways that are oppositely regulated in H_2_O and Abx groups. Note *X*-axis is *z*-score; all pathways in all groups are significant (FDR-corrected *p* < 0.05). ***G***, Cloud diagrams of proteins in the “synaptogenesis signaling pathway”. Each diagram presents the same configuration of proteins with differences between groups indicated by the colored protein nodes. (***H***) Transcription factors predicted to be upstream from downregulated proteins (*left*) and upregulated proteins (*right*). Full proteomics comparisons, pathway analyses, and predicted transcription factors are available in Extended Data Tables 6 - 1-7.

We previously found that microbiome knockdown altered the effect of repeated morphine on transcriptional control in the NAc (Hofford et al., 2021b). Knowing that microbiome knockdown has such a robust effect on transcriptional responses to opioids and that exposure to drug-related cues after a period of abstinence contributes to relapse (Nicolas et al., 2022), the current study examined how microbiome depletion alters the proteome in the NAc at a critical time point after a fentanyl-seeking test (Fig. 6*A*). Both Abx treatment and fentanyl self-administration affected the proteomic landscape but rats in the Abx-IncFR group had the most differentially expressed proteins compared to H_2_O-Sal controls (Fig. 6*B*, full lists of differentially regulated proteins included as Extended Data Table 6-1). Since behavioral differences were strongest between H_2_O- and Abx-IncFR groups, pathway analysis focused on protein expression differences between these groups. A subset of pathways were uniquely predicted in either H_2_O or Abx-IncFR including pathways “regulation of presynaptic cytosolic calcium” in H_2_O-IncFR and “small molecule binding” in Abx-IncFR. (Extended Data Table 6-2). However, most of the top terms associated with substance use disorders (SUD) were present in both groups (Fig. 6*C*), including the predicted upregulated pathways: “amphetamine addiction”, “cocaine addiction”, and “dopaminergic synapse”.

10.1523/ENEURO.0388-23.2023.t6-1 Table 6-1 Full matrix of pairwise proteomics comparisons with fold change and statistics. Download Table 6-1, XLS file.

10.1523/ENEURO.0388-23.2023.t6-2 Table 6-2 Full G:Profiler analysis of significantly regulated pathways in each pairwise comparison. Download Table 6-2, XLS file.

Given the overlap in predicted pathways, we performed pathway analysis on shared proteins that were up- or downregulated in all fentanyl administering groups and were unchanged in Abx-Sal. There were 3 shared downregulated proteins and 20 shared upregulated proteins (Fig. 6*E*). Notably, several proteins that have been implicated in the actions of other drugs of abuse (Przewłocka et al., 1996; Caputi et al., 2014) were upregulated in all fentanyl groups such as PDYN, which had the highest fold change difference in every comparison, and SC6A3, commonly known as the dopamine transporter. No pathways were predicted from downregulated proteins, but several pathways were predicted from upregulated proteins (Extended Data Table 6-3) including the SUD-related terms: “alcoholism”, “amphetamine addiction”, and “opioid signaling” (Fig. 6*D*).

10.1523/ENEURO.0388-23.2023.t6-3 Table 6-3 List of proteins that overlap in all fentanyl administering groups and associated pathways. Download Table 6-3, XLS file.

Using Ingenuity Pathway Analysis, which provides directional z-scores derived from total lists of differentially regulated proteins and their fold change from control, several pathways were significantly predicted to be regulated in opposite directions in H_2_O rats vs Abx rats. Among them, “memory” and “synaptogenesis signaling pathway” were predicted to be downregulated in Abx groups and upregulated in H_2_O groups (Fig. 6*F*, full list of IPA terms as Extended Data Tables 6-4 and 6-5). These pathways were strongly regulated by microbiome status, indicating that these pathways might be important in driving the behavioral differences observed in the current study. To visualize the extent of this discrepancy, cloud diagrams depicting proteins within the synaptogenesis signaling pathway were generated (Fig. 6*G*, full list of proteins in Extended Data Table 6-6).

10.1523/ENEURO.0388-23.2023.t6-4 Table 6-4 Full list of Ingenuity Pathway Analysis canonical pathways in fentanyl administering groups. Download Table 6-4, XLS file.

10.1523/ENEURO.0388-23.2023.t6-5 Table 6-5 Full list of Ingenuity Pathway Analysis diseases and biological functions in fentanyl administering groups. Download Table 6-5, XLS file.

10.1523/ENEURO.0388-23.2023.t6-6 Table 6-6 Synaptogenesis signaling pathway gene list and overlapping proteins by group. Download Table 6-6, XLS file.

Finally, upregulated and downregulated protein lists were analyzed using Enrichr database to identify potential transcription factors that might be regulating the changes in protein expression observed. Only A0A815ZU59 and A0A816A7Q7 (gene names *Zmiz1* and *Yy1,* respectively) were significantly predicted to be oppositely regulated between H_2_O-IncFR and Abx-IncFR (Fig. 6*H*, full list of predicted transcription factors as Extended Data Table 6-7). Additional transcription factors were differentially predicted in some groups; notably, MYC was highly downregulated in all Abx groups but not in any H_2_O groups, suggesting that activity of MYC could be modulated directly by the microbiome and could be affecting motivation and fentanyl-seeking.

10.1523/ENEURO.0388-23.2023.t6-7 Table 6-7 Enrichr lists of predicted upstream transcription factors by group. Download Table 6-7, XLS file.

### Short-chain fatty acid supplementation eliminates Abx-driven enhancement motivation for fentanyl

**Figure 7.**
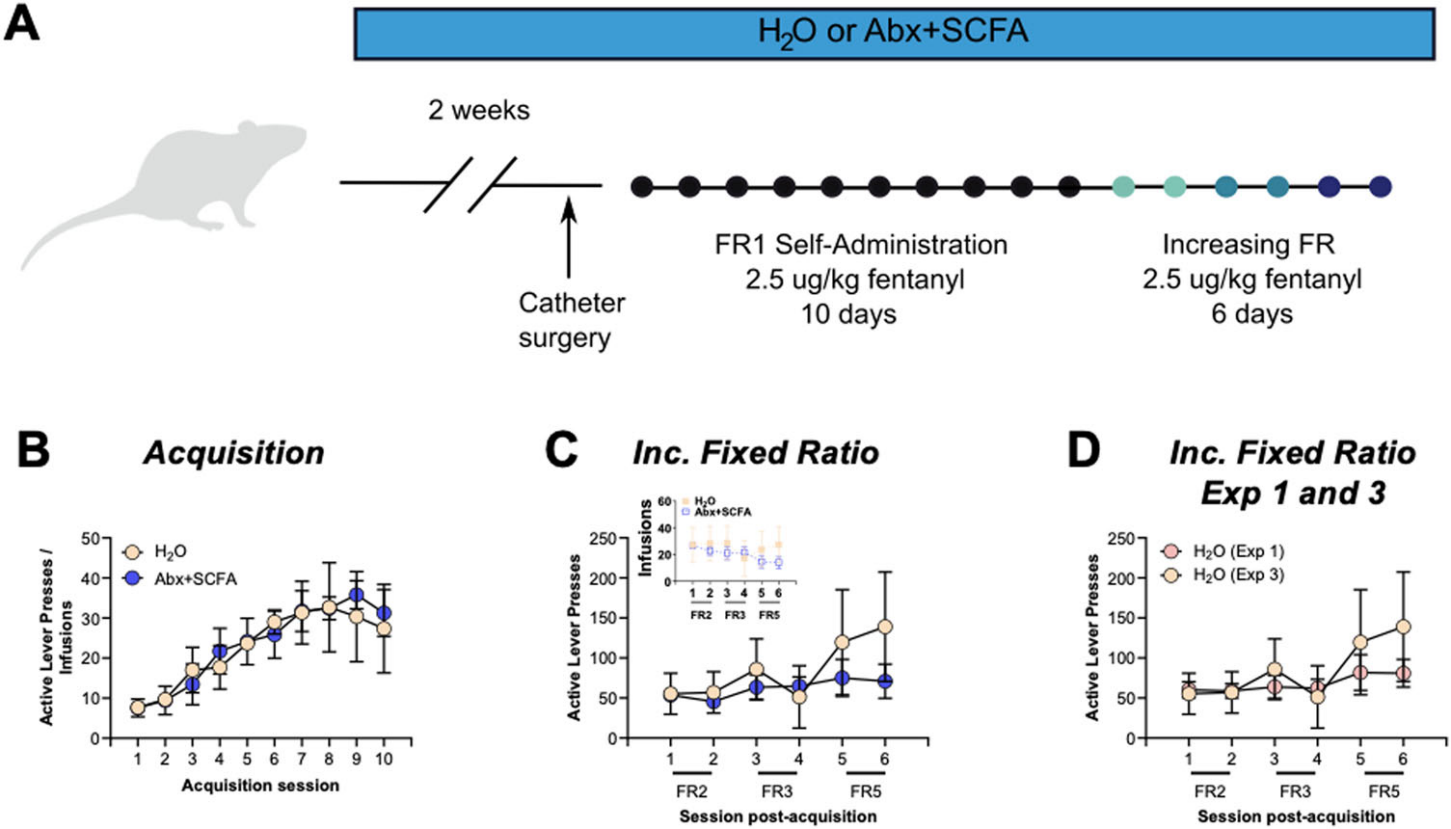
Supplementation with short chain fatty acids (SCFA) reverses behavioral effects of microbiome depletion. ***A***, Experimental schematic for Experiment 3. ***B***, Control and Abx + SCFA treated rats acquired FR1 administration at the same rate. ***C***, When the fixed ratio was increased H_2_O and Abx + SCFA rats maintained similar levels of responding. Inset is infusions earned. ***D***, Progressive ratio breakpoint was not different between H_2_O and Abx + SCFA rats. When comparing the H_2_O control groups from Experiments 1 & 3 there was no difference in responding on increased fixed ratio (***E***) or progressive ratio (***F***) responding. Examination of all groups from Experiments 1 & 3 showed main effect of group with increased responding in the Abx group on the increased fixed ratio (***G***) and progressive ratio (***H***) tasks. *p < 0.05; **p < 0.01.

One of the key pathways for gut microbiome to brain signaling is through production of neuroactive metabolites that can signal to the brain. One such class that has shown significant mechanistic promise is the short-chain fatty acids (SCFA) which are produced by bacteria when fermenting dietary fiber (Dalile et al., 2019). Oral Abx reduces SCFA in rats to virtually undetectable levels (Meckel et al., 2023). Additionally, previous work from our group has shown that repletion of the three main SCFA in animals with their microbiome depleted by antibiotics restores cocaine and morphine conditioned place preference and cocaine seeking to control levels despite having no measurable effect on the microbiome composition (Kiraly et al., 2016; Hofford et al., 2021b; Meckel et al., 2023). However, the mechanistic role of SCFA in reversing fentanyl seeking behavior is as yet untested. Here, Experiment 3 tested the effect of combination Abx + SCFA on fentanyl self-administration (Fig. 7*A*). As with previous experiments, combination Abx + SCFA did not alter fentanyl self-administration during acquisition (Fig. 7*B*, session: *F*_(2.295,17.85)_ = 7.96, *p* = 0.003; treatment: *F*_(1,8)_ = 0.0099, *p* = 0.92; interaction: *F*_(9,70)_ = 0.17, *p* = 0.99). However, despite still having their microbiome depleted, the Abx + SCFA group exhibited no differences during the increasing fixed ratio phase (Fig. 7*C*, session: *F*_(1.59,12.72)_ = 5.29, *p* = 0.03; treatment: *F*_(1,8)_ = 0.48, *p* = 0.51; interaction: *F*_(5,40)_ = 2.21, *p* = 0.07). Similarly, rats treated with Abx + SCFA had similar breakpoint on the progressive ratio task as H_2_O controls (Fig. 7*D* – Mann Whitney: *U* = 8.5; *p* = 0.72).

While not all potential experimental groups were included in this experiment, there is utility in comparing all three main groups here. To do this, we first verified that there were similar levels of responding for the H_2_O control groups between Experiments 1 & 3. On increased FR responding there was no effect of experiment (Fig. 7*E* - *F*_(1,7)_ = 0.26, *p* = 0.62) or day x experiment interactions (*F*_(5,35)_ = 1.78; *p* = 0.14), but there was the expected main effect of day (*F*_(1.696,11.87)_ = 5.29; *p* = 0.027). Similarly, on the progressive ratio task, the two H_2_O groups had nearly identical means for breakpoint (Fig. 7*F* – *U* = 8; *p* = 0.85). Given this, we then analyzed responses for all treatment groups using all H_2_O rats from Experiments 1 & 3 compared to the Abx rats from Experiment 1 and the Abx + SCFA rats from Experiment 3. Here, we see that the Abx animals show increased responding on the increased fixed ratio task compared to both H_2_O and Abx + SCFA groups (Fig. 7*G* – Treatment: *F*_(2,20)_ = 3.64, *p* = 0.047; Day: *F*_(1.791,35.47)_ = 21.07, *p* < 0.0001; Treatment x Day: *F*_(10,99)_ = 3.83, *p* = 0.0002). Similarly, when comparing the three groups on the progressive ratio task, there is a main effect on a Kruskal-Wallis test with significant post-hoc differences between Abx and both other groups (Fig. 7*H* – KW Statistic = 10.38, *p* = 0.006; Dunn’s post-hoc test: H_2_O v Abx - *p* = 0.035; H_2_O v Abx + SCFA – *p* > 0.999; Abx v Abx + SCFA – *p* = 0.007). Taken together, these analyses indicate that SCFA supplementation is sufficient to reverse the behavioral effects of Abx observed in the current study. Together with past data, this strongly suggests that Abx exerts its effects on fentanyl self-administration by reducing levels of SCFA produced by the microbiome.

## Discussion

These studies provide evidence that the microbiome influences opioid reinforcement in a translationally relevant model of opioid use. By utilizing self-administration, we show that microbiome knockdown increases motivation for fentanyl, enhances sensitivity to the reinforcing properties of fentanyl, and increases fentanyl-seeking (Figs. 1, 3). Previously, we observed that mice with a depleted microbiome have reduced morphine conditioned place preference at higher doses of morphine (Hofford et al., 2021b). While there are several experimental differences between the current work and the previous report (e.g., species, opioid, and behavioral test), the morphine CPP results and the self-administration data can generally be explained by the same behavioral mechanism. As observed in Experiment 2 (Fig. 3), rats show enhanced sensitivity to the rewarding effects of fentanyl- demonstrated by a leftward shift in the peak of the dose-response curve. While involving different types of learning (instrumental vs associative), both lever pressing for infusions during self-administration and conditioned place preference (Koek, 2016) are dose-dependent and the dependent variable-dose relationship is shaped as an inverted U. Thus, a *reduction* in high dose morphine place preference could be explained by a leftward shift in the CPP dose-response curve. In line with our results, other laboratories have described a similar enhancement of lower-dose morphine’s effectiveness in tests of antinociception after microbiome knockdown (Kang et al., 2017).

As observed previously, Abx drastically reduced levels of bacterial diversity and significantly shifted the microbiome. Notably, alterations in multiple antibiotic-sensitive genera negatively correspond with fentanyl intake (Fig. 5). Four genera (*Ruminococcus, Butyricicoccus, Lachnospiracae,* and *Anaerotignum*) were significantly reduced in Abx-treated rats, suggesting that lower levels of these bacteria (whether induced by oral Abx or occurring naturally in the H_2_O-treated rats) could be responsible for greater fentanyl intake at higher fixed ratios. While the relationship between genus abundance and fentanyl intake is a correlation and directionality of effect cannot be determined with certainty, it is possible that a reduction in these genera could increase motivation for fentanyl. Interestingly, *Ruminococcus, Butyricicoccus,* and *Lachnospiraceae* are all within the Clostridia class of bacteria; species within this class are producers of SCFA (Louis and Flint, 2017). Interestingly, we do not see effects of fentanyl treatment on the microbiome composition (Fig. 4). Our previous study found that repeated morphine did not alter microbiome composition in mice (Hofford et al., 2021b), but there are others that have shown effects of morphine on the mouse microbiome (Wang et al., 2018; Zhang et al., 2021). Ultimately, the effect of opioids on the composition of the microbiome remains unclear, but there are likely effects related to dose, frequency, route of administration, and specific opioid used.

Additionally, we performed proteomic analysis on NAc samples from rats that self-administered fentanyl or saline in Experiment 1. With the inclusion of saline controls (H_2_O-Sal and Abx-Sal) as well as H_2_O and Abx groups that self-administered the same amount of fentanyl (H_2_O-FR1 and Abx-FR1) and H_2_O and Abx groups that differed in their fentanyl intake (H_2_O-IncFR and Abx-IncFR), the design of this experiment allowed us to separate protein expression differences driven by Abx alone from protein expression alterations that were driven by differences in fentanyl administration secondary to microbiome depletion. From this we see that the greatest number of proteins were altered in the Abx-IncFR group with a relatively modest effect of Abx treatment alone or in the FR1 groups (Fig. 6*B*). This suggests an important interaction of the microbiome manipulation with the behavioral paradigm employed and potentially the amount and schedule of the reinforcers earned.

To examine the changes in the proteome in Abx treated and microbiome intact animals we next performed pathway analysis on the IncFR groups. Here we found that a number of pathways were regulated in similar directions, but with differing magnitudes between the Abx and H_2_O groups (Fig. 6*C*). However, there were also a number of behaviorally relevant pathways that were significantly regulated in divergent directions by H_2_O and Abx regardless of self-administration paradigm, suggesting that processes related to pathways such as memory or synaptogenesis signaling may underlie the behavioral effects of Abx (Fig. 6*F*). Within the synaptogenesis signaling pathway, all groups showed a significant upregulation in GRP2 and a downregulation of ADCY5. However, Abx-IncFR rats overall had more differentially regulated proteins, the majority of which were downregulated, as observed in Figure 6*G*. The upregulation of proteins involved in synaptogenesis signaling found in H_2_O rats is in line with recent work showing that abstinence from fentanyl exposure increases the number of silent synapses in the NAc (Panopoulou and Schlüter, 2022). Interestingly, unlike the H_2_O rats, Abx rats showed a downregulation in this pathway, despite the increase in fentanyl intake at higher FRs. This intriguing finding suggests that microbiome depletion produces different synaptic adaptations in response to fentanyl that might actually enhance motivation for drug administration. Additionally, we identified several proteins that were upregulated in all fentanyl-administering groups regardless of microbiome status including PDYN and the dopamine transporter- proteins not previously linked to fentanyl but implicated in psychostimulant relapse (Przewłocka et al., 1996; Caputi et al., 2014). Finally, we identified MYC as a potential transcription factor of interest as it is predicted to be a driver of transcription for proteins downregulated in both Abx groups (Fig. 5*H*). While MYC is most commonly associated with its function as an oncogene (Vita and Henriksson, 2006), there are numerous studies suggesting a role for its function in brain development, normal brain function, and multiple non-cancer brain pathologies (Ferrer and Blanco, 2000; Pelengaris et al., 2002; Dang et al., 2006; Lee et al., 2011; Marinkovic and Marinkovic, 2021). While research on MYC functions in the adult striatum is relatively limited, single-cell sequencing datasets suggest robust expression in myriad cell types including microglia, astrocytes, and *Adora2a* expressing medium spiny neurons (Dropviz.org). Related to substance use, previous studies have found that MYC is induced by repeated methamphetamine exposure (Thiriet et al., 2001), and that MYC is involved in epigenetic regulation of kappa opioid receptors (Flaisher-Grinberg et al., 2012). Future work from our group will examine this as a potential readout of gut-brain signaling in opioid use.

Importantly, while a number of previous manuscripts have identified the microbiome as playing a role in regulation of chromatin structure and gene expression (Erny et al., 2015; Hoban et al., 2016, 2018; Thion et al., 2018; Chu et al., 2019), these are the first studies that we are aware of to look at microbiome to brain signaling effects on the proteomic landscape in models of neuropsychiatric disease. In our own previous work we found that microbiome depletion or germ-free status followed by repeated morphine treatment lead to dysregulation of several thousand genes in the NAc – an effect that was an order of magnitude larger than control morphine animals (Hofford et al., 2021b; Sens et al., 2023). Here, we find that microbiome depletion and fentanyl administration, particularly on the IncFR pathway, led to robust changes in protein expression in the brain, but much less dramatic than our previous work. Importantly, this manuscript utilizes fentanyl rather than morphine as the opioid of choice, and in our previous work the molecular assessment was performed 24 h after the final injection, while these studies examined protein changes 3 weeks after rats’ last self-administration day. This suggests that the effects of microbiome depletion may lead to long-lasting changes in the NAc proteome. However, future work will have to examine this in more detail.

Finally, we performed experiments to determine if supplementation of the microbiome-derived SCFA metabolites to rats with an Abx depleted microbiome could reverse the behavioral effects. Here, we see that Abx + SCFA rats perform at levels similar to H_2_O controls on all measures (Fig. 7*B–D*). When all three experimental groups are compared the Abx only group is increased in responding on increased FR and progressive ratio tasks (Fig. 7*G*,*H*). These results are in line with our previous work showing mechanistic reversal of microbiome depletion by repleting the SCFA compounds. In our original manuscript on this topic we saw that increased conditioned place preference for cocaine caused by Abx was reversed by SCFA supplementation (Kiraly et al., 2016). Similarly, Abx induces reduced CPP for morphine which was reversed with SCFA supplementation (Hofford et al., 2021b). More recently, in a model of cocaine self-administration and reinstatement we found that SCFA supplementation reversed the increases in both drug taking and seeking (Meckel et al., 2023). Taken together, these findings place the SCFA as a consistent molecular mechanism that can reverse negative effects of microbiome depletion on behavior. Future work to determine the precise molecular mechanisms of these effects will be important for moving this line of work forward.

These studies were performed in male subjects and can only be generalized to this sex. Because prior studies have suggested there are moderate baseline differences in microbiome composition between males and females (Jaggar et al., 2020; Valeri and Endres, 2021) and behavioral outcomes after disruption to the microbiome often differ in sex-dependent ways (Shobeiri et al., 2022), we decided to focus on fully characterizing the effects of microbiome disruption on males first. A separate study will have to be conducted to determine how Abx influences fentanyl self-administration outcomes in females.

This set of studies examines the behavioral and molecular consequences of oral Abx, concluding that microbiome knockdown enhances fentanyl self-administration under increasing FR and low-dose conditions as well as enhances fentanyl-seeking. Abx exposure also alters the NAc proteome in response to fentanyl and identifies functional pathways that might underlie changes in fentanyl self-administration. These studies lay the groundwork for future translational studies that can be harnessed to curb the OUD epidemic.

## Synthesis

Reviewing Editor: Sam Golden, The University of Washington Decisions are customarily a result of the Reviewing Editor and the peer reviewers coming together and discussing their recommendations until a consensus is reached. When revisions are invited, a fact-based synthesis statement explaining their decision and outlining what is needed to prepare a revision will be listed below. The following reviewer(s) agreed to reveal their identity: Alfred Robison, Foster Olive. Note: If this manuscript was transferred from JNeurosci and a decision was made to accept the manuscript without peer review, a brief statement to this effect will instead be what is listed below.

Dear Authors,

The manuscript has been seen by two reviewers, who both felt that you have sufficiently addressed prior reviewer comments and that your manuscript is suitable for publication in eNeuro with some minor corrections. Overall, the reviwers were enthusiastic about the manuscript.

Please address the following minor comments, at which time the Reviewing Editor will make a final decision following resubmission.

- Line 119 - please define the terms "H2O" and "Abx" at first mention here

- Line 158 - please clarify when food was made available again 24 hrs after removal

- Line 179 - please provide method of euthanasia here

- Lines 180 and 189 - how was catheter patency/failure assessed?

- Line 701 - change "shifts the dose-response curve" to "shifts the fentanyl dose-response curve"

- While this is a minor issue of semantics, the authors alternate between use the term "knockdown" and "depletion" when referring to the microbiome. The term "knockdown" can imply a <100% reduction while depletion can carry the connotation of complete elimination. The authors should consider being consistent with one term or the other, or provide a rationale for using both terms.

- The text size in Figures 1A, 3A, and 5A is very small and difficult to read. Even more so in the inset of Figure 1C, 1H, 4A-D, and 6C. Please enlarge font to the extent possible to make it more legible.
